# Design, Synthesis, and Biological Evaluation of New Analogs of Aurein 1.2 Containing Non-Proteinogenic Amino Acids

**DOI:** 10.3390/molecules30092050

**Published:** 2025-05-05

**Authors:** Nora Angelova, Ivan Iliev, Veronica Nemska, Tatyana Dzimbova, Nelly Georgieva, Dancho Danalev, Emilia Naydenova

**Affiliations:** 1 Department of Organic Chemistry, University of Chemical Technology and Metallurgy, 1797 Sofia, Bulgaria; angelovanora@yahoo.com; 2 Institute of Experimental Morphology, Pathology and Anthropology with Museum, Bulgarian Academy of Sciences, 1113 Sofia, Bulgaria; taparsky@abv.bg; 3 Department of Biotechnology, University of Chemical Technology and Metallurgy, 1797 Sofia, Bulgaria; vnemska@uctm.edu (V.N.); neli@uctm.edu (N.G.); ddanalev@uctm.edu (D.D.); 4 Department Sport, Faculty of Public Health, Health Care and Sport, South-West University “Neofit Rilski”, 2700 Blagoevgrad, Bulgaria; tania_dzimbova@abv.bg

**Keywords:** antimicrobial peptides (AMPs), aurein 1.2, antimicrobial activity, antiproliferative activity, nonproteinogenic amino acids, secondary structure prediction

## Abstract

Extensive use of classical antibiotics has led to the growing emergence of many resistant strains of pathogenic bacteria. To combat this challenge, researchers have turned to the antimicrobial peptides (AMPs). Aurein 1.2 (GLFDIIKKIAESF-NH2) was demonstrated to have broad spectrum bi-functionality against bacterial and cancer cells. The Solid Phase Peptide Synthesis (Fmoc-strategy) was used for the synthesis of new analogs of aurein 1.2. The purity of all compounds was monitored by HPLC, and their structures were proven using mass spectrometry. Cytotoxicity and antiproliferative effects were studied using 3T3 NRU and MTT tests, respectively. The antibacterial activity was estimated against Gram-positive and Gram-negative bacteria using broth microdilution method in concentrations from 0 to 320 µg/mL to determine the minimal inhibitory concentration (MIC) and minimal bactericidal concentration (MBC). The antiproliferative activity test shows that the peptide analog EH [Orn]^8^ has the highest activity (IC_50_ = 44 ± 38 μM) for the three cell lines studied (MCF-12F, MCF-7, and MDA-MB-231). The same compound exhibited good antimicrobial activity. The obtained results reveal that replacement of Lys with non-proteinogenic amino acids can increase both the potency and activity spectra of natural template peptides, making them suitable candidates for new drug development.

## 1. Introduction

In recent years, multi-resistant bacterial pathogens have become a worldwide problem and pose a significant threat to global public health. The forecasts indicate that in the upcoming years, mortality rates will increase to alarming levels. In response, the development of antimicrobial agents with enhanced efficacy and reduced resistance potential is imperative. To combat this challenge, many researchers have turned their attention to antimicrobial peptides (AMPs). They are found in the innate immune system of a wide range of organisms and are identified as the most promising alternative to conventional molecules used nowadays against infections. It was found that antimicrobial and anticancer properties of peptides have common beginnings [1]. They both depend on the total negative charge of the bacterial membrane and the tumor surface [2]. As a result of this discovery, many peptides with proven antimicrobial activities have been tested and showed anticancer effects [3,4,5] Antimicrobial peptides offer diverse mechanisms of action and broad-spectrum activity against different pathogens, including multidrug-resistant bacteria [6,7,8]. Produced from a variety of sources, including amphibian skin secretions, AMPs demonstrate potent antimicrobial effects while exhibiting limited cytotoxicity [9,10,11].

AMPs exhibit a diverse range of activities, including antiviral [12,13], antifungal [14], antiparasitic [15], and anti-inflammatory properties [16], making them attractive candidates for pharmaceutical applications [17,18,19,20,21].

AMPs exert antimicrobial activities primarily through mechanisms involving membrane disruption, so they have a lower likelihood of inducing drug resistance [22,23,24,25]. Currently, a number of AMPs are undergoing clinical and preclinical trials against various infectious diseases [26,27]. Koo et al. [26] list the 36 AMPs (27 clinical and 9 preclinical) with brief information about their origin, structure, mechanism, and development status. For example, Novamycin (34, NP339) is an antifungal peptide which induces membrane disruption of *Aspergillus* spp. and *Candida* spp. Ramoplanin (26, NTI-851) is a macrocyclic glycolipodepsipeptide produced by *Actinoplanes* spp. which exhibits bactericidal activity by blocking the cell wall peptidoglycan synthesis of Gram-positive bacteria. Recently, the phase III clinical study of the peptide was initiated for the oral treatment of vancomycin-resistant *enterococcus* (VRE) colonization, as well as the phase II trial against *Clostridium difficile* [27].

Huan et al. [28] reviewed the progress of research on AMPs, including their classification, mechanism of action, design methods, environmental factors affecting their activity, application status, prospects in various fields, and problems to be solved. Despite the expanding diversity of AMPs, many of them are short peptides with a net positive charge. Furthermore, the presence of hydrophobic residues causes the amphipathic fold in AMPs. The positive charge and hydrophobicity are significant factors in bacterial membrane interaction and membrane disruption, respectively [23,29]. Antimicrobial peptides have therapeutic effects with broad-spectrum activities, but there are some hurdles in using AMPs as clinical candidates such as possible toxicity, lack of stability and relatively high budgets required for manufacturing [30]. This can be overcome by developing shorter and more easily accessible AMPs, named short antimicrobial peptides (SAMPs), containing between two and ten amino acid residues [31]. They are an attractive class of therapeutic agents with high potential for clinical use and possessing multifunctional activities [32,33]. Purposeful design of short synthetic peptides has been successfully used to improve the therapeutic properties of AMPs [7,31,34,35]. A large number of the designed antimicrobial peptides contain Arg, Lys, and Trp residues [31,32,36] since cationic residues favor AMP–membrane interactions and could increase the antibacterial activity of peptides [31,37].

Cancer is the second leading cause of death worldwide, so there is an urgent need for novel anticancer drugs with minimal side effects. Many AMPs also act as anticancer peptides (ACPs) [5,38,39,40,41]. They have the ability to pass through cell membranes and destroy cancer cells as well as bacterial ones. These peptides offer several advantages, including a short interaction timeframe, low toxicity to normal cells, specificity, and good tumor penetration, positioning them as promising candidates for future chemotherapy treatments.

Aurein peptides are a short α-helical peptide, first isolated from the skin secretion of the Australian bell frogs *Litoria aurea* and *Litoria raniformis* [11,42]. They can be classified into five subgroups (aureins-1–5), among which aurein-1 peptides are regarded as the shortest of α-helical peptides with both antimicrobial and anticancer activities. Aurein 1.2, the most studied peptide in the aurein family, has demonstrated a broad spectrum of bi-functionality against bacterial and cancer cells [11,42,43].

Aurein 1.2 (GLFDIIKKIAESF-NH2) is a short 13-residue antimicrobial peptide with molecular mass of 1480 Da and positive (+1) net charge at physiological pH [11,42,43]. Aurein 1.2 exerts cytotoxic activity against several human neoplastic cell lines of leukemia and melanoma as well as the lung, colon, central nervous system, ovarian, prostate, and breast cancer cell lines [43].

Antimicrobial activity of aurein 1.2 ranges between 1 and 16 μg/mL in terms of minimum inhibitory concentrations (MICs) against both reference and clinical strains of Gram-positive bacteria such as *Enterococcus faecalis*, *Staphylococcus aureus*, or *Streptococcus pyogenes*. Moreover, a synergistic effect between aurein 1.2 and minocycline/clarithromycin against those bacteria has also been reported [44].

Lorenzón et al. [45] explore the effects of dimerization on the structure and biological activity of the AMP aurein 1.2. They found that antimicrobial activity against bacteria and yeast decreased with dimerization. However, dimeric peptides promoted the aggregation of *C. albicans*. On the other hand, in vitro studies demonstrated that aurein 1.2 exhibits a low antimicrobial activity against *Escherichia coli* and *Pseudomonas aeruginosa* (MIC values for both strains are 256 μg/mL) and moderate activity against *Candida albicans* strains (MIC value of 32 μg/mL) [45].

Aurein 1.2 does not adopt any secondary structure in aqueous media. However, after incorporation into the lipid membrane, it assumes an α-helical geometry. The membrane interactions of the antimicrobial peptide aurein 1.2 were studied using a range of biophysical techniques to determine the location and the mechanism of action in two model membranes that mimic characteristics of eukaryotic and prokaryotic membranes, respectively [46]. Fernandez et al. demonstrated that aurein 1.2 exerts disruptive activity via a carpet-like mechanism against both neutral and anionic model membranes, with a greater affinity towards the latter [46]. Furthermore, significant alternation of lipid components between the negatively charged model membrane leaflets due to aurein 1.2 binding has also been demonstrated [47]. In their in vivo studies, Laadhari et al. suggest that antimicrobial activity of aurein 1.2 is not only an effect of membrane destabilization and/or disruption, but that interactions between the peptide and bacterial teichoic acid and lipoteichoic acid should also be considered [48].

It is known that (KLAKLAK)_2_ is another cell-penetrating antimicrobial peptide that also shows anticancer activity [49,50,51,52,53,54,55]. This sequence is always used as a membrane-disrupting (pro-apoptotic) domain in anticancer peptide design. Therefore, Liao et al. [43] selected the cell-penetration regions -IIKK- and -KLA- as lysine-based modifications, providing analogs with broad-spectrum multifunctionality.

Taking into account the importance of Lys residues for biological activity, lysine-based modifications in the structure of aurein 1.2 were performed in this study, with the hope that the substitutions made would further increase the antimicrobial and anticancer potencies of the peptides. Herein, we report the synthesis, antimicrobial, and anticancer potencies of the 7 newly synthesized peptides. Thus, the molecule of aurein 1.2 was modified by replacing the lysine residues at position 7 and 8, sequentially and simultaneously with the non-proteinogenic amino acids Orn, 2,4-diaminobutyric acid (Dab), or 2,3-diaminopropanoic acid (Dap). So, the main aim of the current study was to investigate the importance of the Lys and distance of the side-chain amino group from the peptide backbone on biological activity.

## 2. Results

### 2.1. Synthesis and Characterization of Target Compounds

**Scheme 1:**
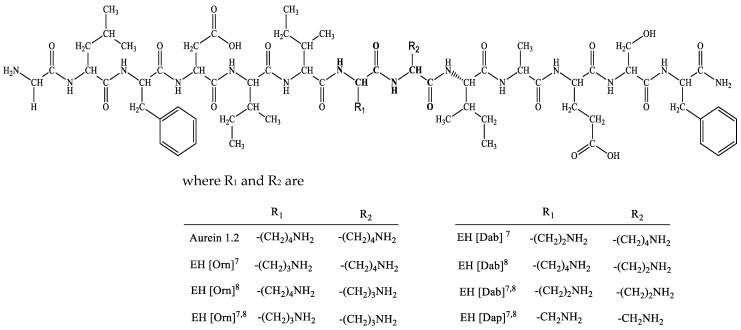
Chemical structures of aurein 1.2 and its analogs.

Aurein 1.2 is a short antimicrobial peptide containing 13 amino acids with an amidated C-terminus. In this study, aurein 1.2 was chosen as a template for rational modification to achieve more potent biologically active peptides. Towards the goal of improvement of activity, seven analogs of aurein 1.2 were designed and synthesized as C-terminal amides with the following general structure:

Gly^1^-Leu^2^-Phe^3^-Asp^4^-Ile^5^-Ile^6^-Xxx^7^-Xxx^8^-Ile^9^-Ala^10^-Glu^11^-Ser^12^-Phe^13^-NH_2_

where Xxx is Orn, Dab or Dap Lys residues at position 7 and 8 were replaced sequentially and simultaneously with the mentioned non-proteinogenic amino acids (Scheme 1).

**Scheme 2:**
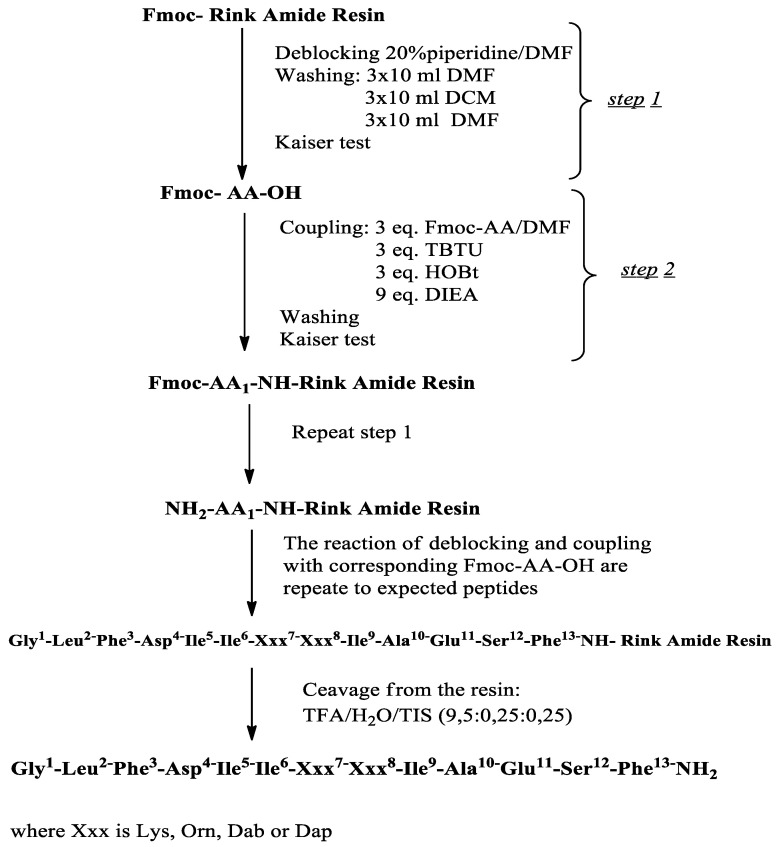
Synthetic route for obtaining aurein 1.2 and its analogs.

**Scheme 3:**
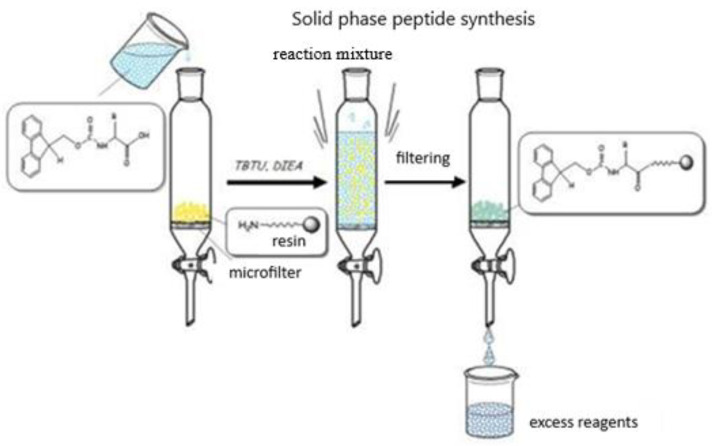
Manual solid-phase peptide synthesis.

All compounds were synthesized using the conventional solid-phase peptide synthesis (SPPS), Fmoc/Ot-Bu strategy. Rink-amide MBHA resin was used as a solid-phase carrier to obtain C-terminal amides (Scheme 2 and Scheme 3).

The peptide purity was monitored on a RP-HPLC. The LC/MS spectra were recorded on a LTQ XL Orbitrap Discovery instrument. A modular circular polarimeter (Anton Paar Opto Tec GmbH, Seelze, Germany) was used in carrying out the optical rotations of the peptides.

Analytical data for newly synthesized peptides are summarized in Table 1. The molecular masses of the compounds are in accordance with the theoretical weights, demonstrating that all peptides were successfully obtained.

### 2.2. Secondary Structure Prediction

**Figure 1:**
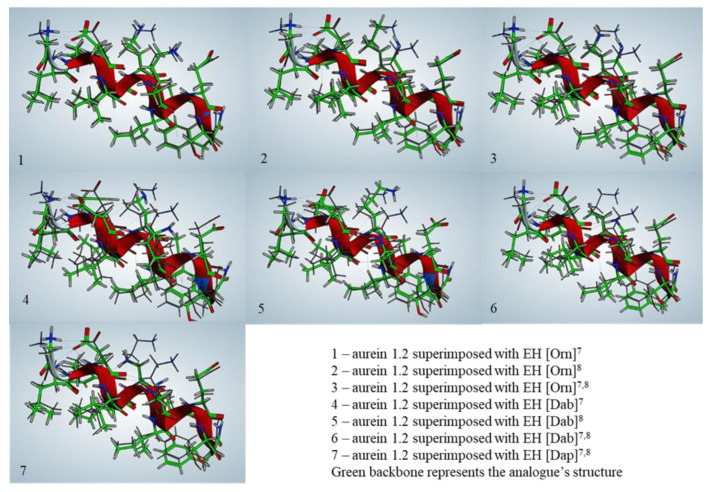
The representation of superimpositions of each analog with aurein 1.2.

The secondary structures of analogs of aurein 1.2. were predicted by using the software Avogadro (an open-source molecular builder and visualization tool, Version 1.20 [56]), and Molecular Operating Environment (MOE) [57] was used for structure analysis. The structure of aurein 1.2 was obtained from the RCSB PDB (id: 1vm5) [58]. After changes in the amino acid sequence with the corresponding non-proteinogenic amino acid at positions 7 and 8 (Orn, Dab, and Dap), the structures of the seven new analogs were generated. These structures were optimized, and the energies of the obtained conformations were calculated (Table 2). The modelled structures, superimposed along their backbone N, α-C, and carbonyl-C atoms with the structure of the aurein 1.2, are shown in Figure 1. They clearly show an α-helical conformation along the length of the peptide. The Root Mean Square Deviation (RMSD) was also calculated.

**Figure 2:**
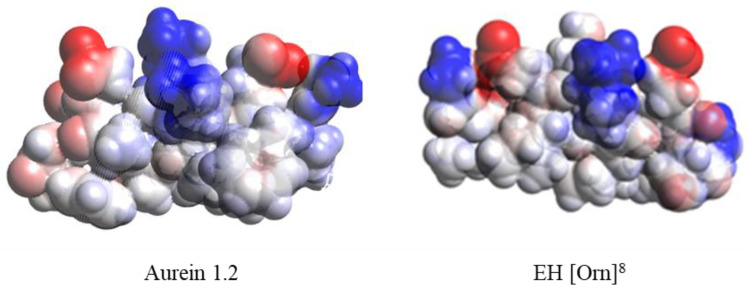
Potential surfaces of aurein 1.2 and EH [Orn]^8^. Hydrophobic grooves bordered by positive charges have a high membrane-perturbation potential.

Potential surfaces of aurein 1.2 and its analogs were determined by computer modeling by Software MOE2022.02 (Molecular Operating Environment). Hydrophobic grooves bordered by positive charges have a high membrane-perturbation potential (Figure 2).

### 2.3. Secondary Structures of the Peptides Studied by CD

**Figure 3:**
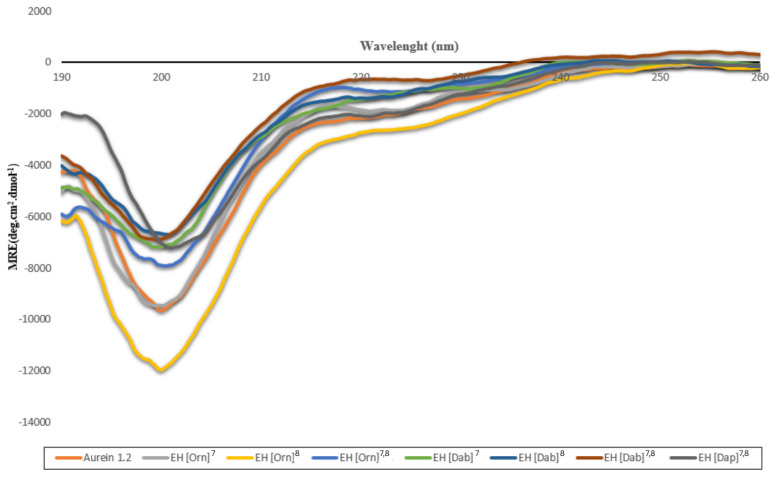
CD spectra of aurein 1.2 (orange); EH [Orn]^7^ (gray); EH [Orn]^8^ (yellow); EH [Orn]^7,8^ (blue); EH [Dab]^7^ (green); EH [Dab]^8^ (dark blue); EH [Dab]^7,8^ (brown); EH [Dap]^7,8^ (dark gray) in deionized water solution at pH 7.

The secondary structure confirmation was performed in a circular dichroism study. Peptides were dissolved in deionized water (pH 7) at a concentration of 100 μM. In this, all the peptides show negative bands in the region between 200 and 210 nm (Figure 3). The obtained CD spectra clearly show that the peptides tended to form random coil conformation and α-helical structures. Aurein 1.2 (Reference Peptide, orange curve) displays distinct minima at 208 nm and 222 nm, confirming a stable α-helical structure. The helical structure suggests it retains some conformational stability in aqueous environments, though it may be further stabilized in membrane-mimicking conditions. The peptide EH [Orn]^8^ (yellow curve) exhibits a pronounced negative peak near 200 nm, indicative of a predominantly random coil conformation. This suggests that this peptide does not form stable secondary structures in water, likely due to weak hydrophobic interactions or insufficient intra-peptide hydrogen bonding. The peptide EH [Orn]^7^ (gray curve) displays strong negative peak near 200–205 nm, indicative of a random coil conformation. The absence of characteristic α-helical minima suggests a lack of stable secondary structure in water. The peptide EH [Orn]^7,8^ (blue curve) displays a broad negative region with no strong α-helical signatures, indicative of a predominantly random coil structure with minor folding tendencies. The intermediate behavior suggests that substitutions at both positions 7 and 8 contribute to structural destabilization.

The peptides EH [Dab]^7^ (green curve), EH [Dab]^8^ (dark blue curve), and EH [Dab]^7,8^ (brown curve) exhibit well-defined negative peaks at 208 nm and 222 nm, characteristic of an α-helical conformation, which indicates a higher degree of structural organization, likely due to amphipathic properties and increased stability from hydrogen bonding. Dual Dab substitutions appear to either maintain or slightly enhance helical stability in water.

The peptide EH [Dap]^7,8^ (dark gray curve) exhibits an intermediate spectrum, suggesting a mixture of α-helix and random coil. The substitution of Lys with Dap leads to partial destabilization compared to Dab-containing peptides but retains more structure than Orn-containing analogs.

### 2.4. In Vitro Safety Testing

**Figure 4:**
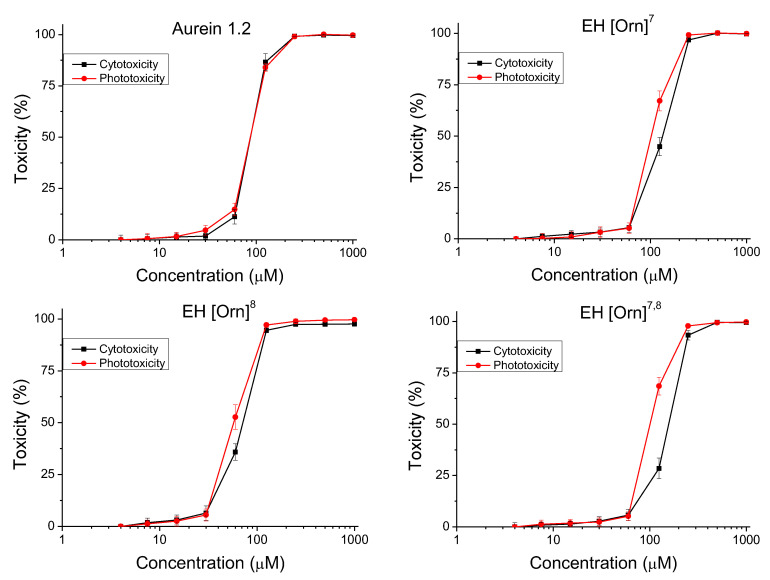

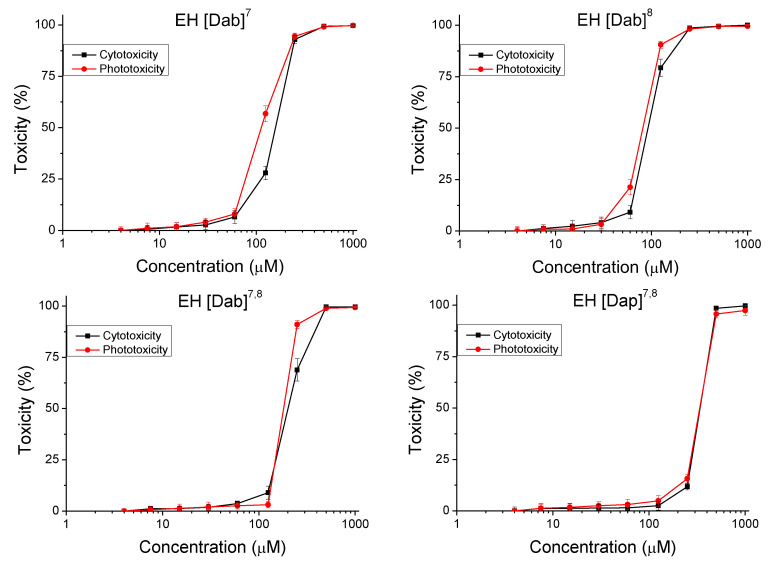
Cytotoxicity/phototoxicity of peptides determined in BALB 3T3 cells. Peptides were tested in two-fold increasing concentrations from 4 to 1000 μM, n = 6.

An in vitro BALB 3T3 Neutral Red Uptake assay was used to determine the cytotoxicity/phototoxicity of the peptides. Cytotoxicity of peptide analogs was calculated as a percentage relative to the negative control. The resulting sigmoidal dose–response curves describing cyto- and phototoxicity are presented in Figure 4. The CC_50_ (50% cytotoxic concentration) values were calculated by nonlinear regression analysis and are presented in Table 3. Increased cytotoxicity compared to the control peptide (aurein 1.2) was observed only at EH [Orn]^8^ with an IC_50_ value = 75.62 ± 3.33 µM. The remaining peptide analogs have lower cytoxicity. The peptides with the lowest cytoxicity are EH [Dap]^7,8^ (CC_50_ = 360.00 ± 2.52 μM) and EH [Dab]^7,8^ (CC_50_ = 201.81 ± 10.11 μM). Photo irritation factor (PIF) was used to assess the phototoxic potential of the peptide analogs. The PIF factor represents the ratio between the CC_50_ values of dark (non-irradiated or—Irr) and irradiated (+Irr) microplates, PIF = (CC_50_ − Irr)/(CC_50_ + Irr). The PIF factor for all peptides studied was lower than 2, indicating a high level of photo safety. 

### 2.5. In Vitro Antiproliferative Activity

**Figure 5:**
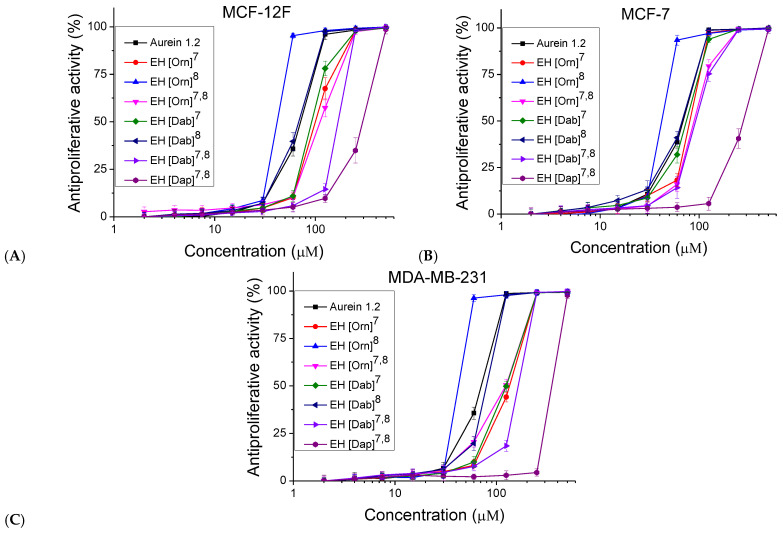
Antiproliferative activity of peptide analogs in the: (**A**) normal MCF-12F cell line (in vitro model of healthy tissue). (**B**) MCF-7 (in vitro model of luminal type A breast cancer). (**C**) MDA-MB-231 (in vitro model of basal B-type breast cancer), n = 6.

The peptides were studied for antiproliferative activity with an MTT-dye reduction assay. Cell cultures were incubated with the test peptides in twofold increasing concentrations (from 2 to 500 μM) for 72 h. The results are presented as sigmoidal curves in Figure 5. The IC_50_ values and selectivity index (SI) were presented in Table 4. No antiproliferative effect at peptide concentrations lower than 30 µM were observed. The calculated IC_50_ values for the studied peptide analogs were between 44 and 307 µM. The weakest antiproliferative effect on MCF-12F cells was observed for the peptides EH [Dap]^7,8^ and EH [Dab]^7,8^ with IC_50_ values = 307.96 ± 21.71 μM and 167.93 ± 2.54 μM, respectively.

The MCF-7 cell line is a model of luminal-A type, hormone-dependent breast cancer. In this cell line, significant antiproliferative activity of the studied peptides was observed compared to the triple negative, basal type breast cancer (MDA-MB-231). The highest antiproliferative activity was observed in EH [Orn]^8^ (IC_50_ = 44.38 ± 1.08 µM) and EH [Dab]^8^ (IC_50_ = 67.21 ± 2.57 µM). The peptide analog EH [Dab]^7,8^ exhibits the highest selectivity towards the tumor cell line MCF-7 (SI = 1.82). The peptides showed lower selectivity towards basal-type breast cancer (MDA-MB-231) compared to luminal type A breast cancer (MCF-7).

### 2.6. Antimicrobial Activity

The antimicrobial activity of the 7 new analogs of aurein 1.2 was evaluated by determining the MIC and MBC against Gram-positive strain *Bacillus subtilis* 3562 and Gram-negative strain *Escherichia coli* K12 407. The MIC of the newly synthesized analogs was assessed by applying a broth microdilution method, whereas their MBC was determined by using a spread plate method. All experiments were performed in triplicate. The obtained results are summarized in Table 5.

The results showed that the MIC of the analogs against *B. subtilis 3562* decreased to 80 µg/mL, even 40 µg/mL (for EH [Orn]^8^), compared to the original peptide aurein 1.2 (MIC = 160 µg/mL), which indicates a better antibacterial activity of the newly synthesized peptides. Only the value of MIC for EH [Dab]^7,8^ and EH [Dap]^7,8^ was the same as or higher than the starting compound, respectively (Table 5).

The values of the MBC against *B. subtilis* 3562 were higher than the MIC (MBC = 320 µg/mL) of the newly obtained compounds. The exception was the peptides EH [Orn]^8^, EH [Dab]^7,8^, EH [Dap]^7,8^, and the starting peptide, for which the MBC had not been reported, which means that the derived compounds have a bacteriostatic effect against *B. subtilis* 3562.

The results with Gram-negative strain *E. coli* K12 407 showed that the MIC of most of the newly synthesized peptides increased up to 80 µg/mL and 160 µg/mL for EH [Dab]^7,8^, compared to aurein 1.2. This means that in the case of derivative compounds, the antimicrobial effect is significantly reduced. Only the value of MIC for EH [Orn]^8^ was the same as the starting compound (Table 5).

MBC against *E. coli* K12 407 was not reported, which indicates a very weak bacteriostatic effect of the studied peptides against this strain.

## 3. Discussion

AMPs are regarded as effective weapons in bacterial-related infections and cancer therapy [59]. However, some undesired properties, such as low in vivo efficacy and potential toxicity, have complicated the development of the peptides for clinical use [60]. Many natural AMPs are up to 50 amino acids long. The length of AMPs increases the production costs of AMPs. The solid phase peptide synthesis is more convenient for obtaining peptides that are not too long; therefore, the development of short AMPs with high biological activity is the goal of many researchers.

Some research on aurein 1.2 has demonstrated that this peptide has broad-spectrum antimicrobial and anticancer properties, though these activities were moderate [11,44]. In this study, aurein 1.2 was chosen as a template for rational modification to achieve a more potent biologically active peptide. Taking into account that use of non-proteinogenic amino acids avoids quick elimination by resisting metabolism and the importance of Lys residues for the biological activity of aurein 1.2, we replaced Lys at position 7 and 8, sequentially and simultaneously with the nonproteinogenic amino acids Orn, Dab, or Dap.

Moreover, in our previous studies related to the preparation of analogs of the opioid peptide N/OFQ(1–13)NH_2_, it was revealed that the shortening of the Lysine side chain could be a positive modification for biological activity [61]. The new N/OFQ(1–13)NH_2_ analogs exerted strong and naloxone-resistant inhibition of electrically evoked contractions of rat vas deferens. Lys replacement with Orn maintained or even enhanced the inhibitory activity, while replacements with Dab and Dap have the opposite effect [61].

By computer modeling, the structures of designed peptides were optimized and the energies of the obtained conformations were calculated. From the calculated energy of the generated structure (Table 2), it can be seen that in three of the analogs—EH [Orn]^8^, EH [Orn]^7,8^, and EH [Dap]^7,8^—the energy of the resulting α-helical structure is higher than that of aurein 1.2. Since the energy of EH [Dap]^7,8^ is the highest and due to the fact that it has the weakest antiproliferative effect, it most likely does not form a helix and, accordingly, cannot bind to both the tumor and bacterial membranes. In the others, the probability of forming a helical structure is even higher, due to the lower energy. No relationship was found between the energy of the molecules and RMSD. A relationship was found between the energies of the molecules and their antiproliferative activity (Pearson r 0.6, 0.62 and 0.55, but *p* value > 0.05 for MCF-12F, MCF-7, and MDA-MB-231, respectively); however, due to the small number of compounds, it is not statistically significant.

Combining CD spectroscopy results with computational structure predictions provides deeper insights into the secondary structure and stability of these aurein 1.2 analogs:

Aurein 1.2 (Reference Peptide)—CD confirms its strong α-helical structure, in agreement with its known crystallographic structure (PDB ID: 1vm5). Computational analysis shows it has the lowest energy, reinforcing its conformational stability.

Orn-containing peptides (EH [Orn]^7^, EH [Orn]^8^, EH [Orn]^7,8^)—CD suggests they exist as random coils in water. Computational models predict α-helical structures, but with higher energy values than aurein 1.2, indicating reduced stability. Orn substitutions at positions 7 and 8 likely disrupt helical formation.

Dab-containing peptides (EH [Dab]^7^, EH [Dab]^8^, EH [Dab]^7,8^)—CD confirms α-helical structures. Computational analysis indicates lower energy values, supporting their stability. Dab substitutions enhance helicity, making these promising candidates for membrane interactions.

Dap-containing peptide (EH [Dap]^7,8^)—CD suggests an intermediate structure. Computational modeling shows it has the highest energy among all analogs, indicating low stability. Its weak antiproliferative activity supports the hypothesis that it does not form a stable α-helix.

To determine the safety level of aurein 1.2 and its newly synthesized analogs, we performed cytotoxicity and phototoxicity tests on mouse embryonic fibroblasts. For the peptide analogs EH [Dab]^7,8^ and EH [Dap]^7,8^, we observed two to four times lower toxicity compared to the parent peptide aurein 1.2. We did not observe a phototoxic effect for any of the peptides studied. These results indicate an increased level of safety for topical and systemic application of the above-mentioned peptide analogs. From the antiproliferative activity test, we found that the peptide analog EH [Orn]^8^ has the highest activity (IC_50_ = 44 μM) for the three cell lines studied (MCF-12F, MCF-7 and MDA-MB-231). However, this peptide does not show selectivity between normal and tumor cells. Increased selectivity (SI = 1.82) was observed with the peptide analog EH [Dab]^7,8^ against the tumor cell line MCF-7 (luminal type A breast cancer).

In the antimicrobial activity evaluation, among all analogs of aurein 1.2, containing Orn, Dab and Dap substitutions in positions 7 and 8 the EH [Orn]^8^-analog demonstrated the most potent ability for the growth inhibition and bactericidal activity of Gram-positive and Gram-negative bacteria.

According to the results obtained from the determination of MIC and MBC, newly synthesized analogs showed promising antimicrobial activity against *E. coli* K12 407 and *B. subtilis* 3562. The replacement of Lys in position 7 in molecule of aurein 1.2 with the amino acids Orn or Dab leads to an increase in the antibacterial activity of the obtained peptides against the *B. subtilis* 3562. At the same time, the replacement of Lys in positions 7 and 8 with the amino acid Dab retained the antimicrobial activity, whereas the Dap lowered it.

The chemical structural differences in the surfaces between Gram-positive bacteria and Gram-negative bacteria are key factors leading to different mechanisms. It is essential that the thickness of the cell wall also varies between different bacterial species [62].

A very important advantage of the newly synthesized analogs is that the overall charge of the resulting peptide does not change. The hydrophobic and basic residues are favorable factors with respect to negatively charged membranes (Figure 2). Such a structure favors binding to anionic lipids, which can lead to lipid reorganization in bacterial and tumor membranes and thus exert its antiproliferative and inhibitory effect.

## 4. Materials and Methods

### 4.1. Materials

The protected amino acids and Fmoc-Rink Amide 4-methylbenzhydrylamine (MBHA) Resin were purchased from Iris Biotech (Wunsiedel, Germany). All other reagents and solvents were analytical or HPLC (High-Performance Liquid Chromatography) grade and were bought from Valerus (Sofia, Bulgaria)and Iris Biotech (Wunsiedel, Germany). The 4-N,N-dimethyl aminopyridine (DMAP) was obtained from Sigma-Aldrich (Ansbach, Germany). The solvents N,N′-dimethylformamide (DMF) and dichloromethane (DCM) were bought from Valerus (Sofia, Bulgaria). The reagents and solvents were used as purchased without any additional purification or pretreatment.

### 4.2. Peptide Synthesis and Chemical Analysis

The novel analogs of aurein 1.2. were synthesized using the conventional solid-phase peptide synthesis (SPPS), Fmoc (9-fluorenylmethoxycarbonyl)/OBut strategy. Rink-amide MBHA resin was used as a solid-phase carrier to obtain C-terminal amides. TBTU (2-(1H-benzotriazole-1-yl)-1,1,3,3-tetramethyluronium tetrafluoroborate) or DIC (N,N′-diisopropylcarbodiimide) were used as a coupling reagent, with DIPEA (N,N-diisopropylethylamine) as a base or DMAP (4-N,N-dimethylaminopyridine) as catalysts, depending on the condensation agent.

Three-functional amino acids were embedded as follows: N^α^-Fmoc-Lys(Boc)-OH, N^α^-Fmoc-Orn(Boc)-OH, N^α^-Fmoc-Dab(Boc)-OH, N^α^-Fmoc-Dap(Boc)-OH, Fmoc-L-Ser(tBu)-OH, Fmoc-L-Glu(OtBu)-OH*H_2_O, Fmoc-L-Asp(OtBu)-OH.

The syntheses were carried out in a 20 mL manual SPPS reaction vessel. The coupling reactions are carried out for 1.5–2 h using the molar ratios of reagents as follow: amino acid/TBTU/1-hydroxybenzotriazole (HOBt)/N,N-diisopropylethylamine (DIPEA)/resin a molar ratio 3/3/3/9/1 or amino acid/N,N′-diisopropylcarbodiimide (DIC)/resin a molar ratio 3/3/1, with a catalytic amount of DMAP, respectively.

After the reaction time and washing according to the protocol (3 × 1 min with 10 mL DMF, 3 × 1 min with 10 mL DCM and 3 × 1 min with 10 mL DMF), a Kaiser test is performed. If the condensation has occurred completely, the Fmoc-protecting group is deblocked.

The αN-Fmoc-protecting group was removed during every step by treatment with 10 mL 20% piperidine solution in DMF. Both deprotection and condensation reactions were monitored by Kaiser test.

The cleavage of the synthesized peptide from the resin was done, using a mixture of 95% trifluoroacetic acid (TFA), 2.5% triisopropylsilan (TIS) and 2.5% water. The peptide was obtained as a filtrate in TFA and precipitated with cold dry ether. The precipitate was filtered, dissolved in water and lyophilized to obtain the crude peptide. The peptide purity was monitored on a RP-HPLC XTera C18 3.5 μm (125 × 2.1 mm) (Waters Co. Milford, MA, USA) column, flow 200 μL/min, using a linear binary gradient of phase B from 10% to 90% for 15 min (phase A: 0.1% HCOOH/H_2_O; phase B: 0.1% HCOOH/Acetonitrile) The compounds were checked by electrospray ionization mass spectrometry. The Liquid chromatography–mass spectrometry (LC/MC) spectra were recorded on a Linear Ion Trap Mass Spectrometer (LTQ XL), Thermo Fisher Scientific, Waltham, MA, USA. The optical rotation was measured on automatic standard polarimeter Polamat A, Carl Zeis, Jena (Jena, Germany).

The melting points were determined on a standard Kofler apparatus and are wereuncorrected. A modular circular polarimeter (Anton Paar Opto Tec GmbH, Seelze, Germany) was used in carrying out the optical rotations of the peptides. The analytical data for the synthesized peptides is shown in Table 1.

The software Avogadro (an open-source molecular builder and visualization tool, Version 1.20 [56]) and Molecular Operating Environment (MOE) [57] were used for structure analysis of the newly synthesized analogs of aurein 1.2. The structure of aurein 1.2 was obtained from the RCSB PDB (id: 1vm5) [58]. After changes in the amino acid sequence with the corresponding amino acid at positions 7 and 8 (Orn, Dab, and Dap), the structures of the seven new analogs were generated. These structures were optimized, and the energies of the obtained conformations were calculated (Table 2). For the statistical analysis, GraphPad Prism 3.0 Software was used.

CD spectra were recorded using a Jasco J-815 spectropolarimeter (JASCO Corporation, Tokyo, Japan). Peptides were dissolved at a concentration of 100 μM in deionized water at pH 7. Measurements were conducted in a 1 mm path length quartz cuvette at 20 °C, covering a wavelength range of 190–260 nm. The spectra were obtained in continuous scanning mode with a response time of 1.0 s, a step size of 0.1 nm, and a bandwidth of 2 nm. To enhance the signal-to-noise ratio, each spectrum was recorded as the average of three independent scans. Baseline correction was applied by subtracting the background spectrum of the solvent from the peptide spectra.

### 4.3. Cell Cultures

The human breast epithelial cells (MCF-12F), mouse embryonic fibroblasts (BALB 3T3 clone A31), basal B-type breast cancer (MDA-MB-231), and luminal A breast cancer (MCF-7) were used in in vitro experiments. Cells were purchased from the American Type Cultures Collection (ATCC, Manassas, VA, USA). Cell cultures were growth in culture medium DMEM—high glucose (4500 mg/L glucose), 10% FBS, and antibiotics (Sigma-Aldrich, Schnelldorf, Germany). Cell cultures were incubated at 5% CO_2_, 37 °C, and 95% humidity in plastic flasks (25 cm^2^ and 75 cm^2^) (Biologix, Lenexa, KS, USA).

### 4.4. Safety Testing

Mouse embryonic fibroblasts (BALB 3T3) were used to perform the cytotoxicity and phototoxicity tests. The tests were performed by OECD Guidelines for the Testing of Chemicals, Section 4, Test No. 432. Cells were plated at 1 × 10^4^ cells/well in 96-well plates. Cells were incubated in a thermostat for 24 h. Then, the test peptides were added at various working concentrations (4–1000 μM). In the phototoxicity test, 96-well plates were irradiated with a dose of 2.4 J/cm^2^ using a solar light simulator Helios-iO (SERIC Ltd., Tokyo, Japan). Cell viability was measured using the BALB 3T3 neutral red uptake test [63]. Cytotoxicity is presented as % relative to the negative control.

### 4.5. Antiproliferative Activity

The antiproliferative activity of the peptides was determined in normal epithelial cells (MCF-12F) and tumor cells (MCF-7 and MDA-MB-231) by MTT-assay [64]. Cells were plated 1 × 10^3^ cells/100 µL/well of 96-well plates and incubated in a thermostat under standard conditions for 24 h. Cells were then incubated with the tested peptide analogs for 72 h in different concentrations (from 2 to 500 µM). The optical density of the formazan was measured at λ = 540 nm using a microplate reader. Antiproliferative activity was expressed as % from negative control. The IC_50_ values (50% inhibitory concentration) were determined by nonlinear regression analysis. The statistical analysis of the results was performed using one-way ANOVA followed by Bonferroni’s post hoc test by GraphPad Prism 8 software (San Diego, CA, USA).

### 4.6. Antimicrobial Assays

#### 4.6.1. Test Microorganisms and Culture Conditions

*Bacillus subtilis* NBIMCC 3562 and *Escherichia coli* NBIMCC K12 407 were used as test microorganisms and were purchased from the National Bank for Industrial Microorganisms and Cell Cultures (NBIMCC, Sofia, Bulgaria). Exponential cultures were obtained in Nutrient broth (NB, HiMedia, Mumbai, India) for *B. subtilis* NBIMCC 3562 and Luria-Bertani (LB, HiMedia, Mumbai, India) for *E. coli* NBIMCC K12 407, after cultivation in an incubator shaker ES-20/60 (Biosan, Latvia) at 30/37 °C for 24 h. Then, the turbidity of the microbial cultures was adjusted to the 0.5 McFarland standard using a Grant Bio DEN-1B Densitometer (Grant Instruments, Royston, UK).

#### 4.6.2. Determination of Minimum Inhibitory and Minimum Bactericidal Concentrations

The antimicrobial activity of the obtained aurein 1.2 analogs was evaluated using the broth microdilution method, recommended by the Clinical and Laboratory Standards Institute (CLSI) [65], to determine the minimum inhibitory concentration (MIC) of the peptides against *B. subtilis* NBIMCC 3562 and *E. coli* NBIMCC K12 407. All peptides were previously dissolved in 10% EtOH/H_2_O, and their 10 mg/mL stock solutions were stored at −20 °C. Bacterial cultures, previously adjusted to 0.5 McFarland standard, were incubated in the presence of different peptide concentrations (0–320 µg/mL) in 96-well polystyrene microplates (Deltalab S.L., Barcelona, Spain) at 30/37 °C for 24 h. The lowest concentration of each peptide, which inhibited bacterial growth, was considered as MIC. In addition, a drop of 20 µL of the 24 h inhibitory concentration test samples was spotted on the corresponding solid media to determine the minimum bactericidal concentration (MBC). The MBC was taken as the lowest concentration of each peptide which resulted in more than 99.9% reduction of the initial inoculum. All experiments were performed in triplicate.

## 5. Conclusions

In conclusion, in this study, a broad-spectrum multi-biofunctional peptide, aurein 1.2, was chosen as a template for the design and synthesis of analogs with superior activities.

Non-proteinogenic amino acids (Orn, Dab, and Dap), were introduced into the natural template sequence to hopefully increase the peptide’s functionality and selectivity.

The replacement of Lysine at position 7 and 8 sequentially and simultaneously with the mentioned nonproteinogenic amino acids leads to a better anticancer activity. The highest antiproliferative activity was observed with EH [Orn]^8^ (IC_50_ = 44.38 ± 1.08 µM) and EH [Dab]^8^ (IC_50_ = 67.21 ± 2.57 µM). The studied peptides do not exhibit phototoxicity. According to the results obtained from the determination of MIC and MBC, newly synthesized analogs showed promising antimicrobial activity against *E. coli* K12 407 and *B. subtilis* 3562.

The CD spectra clearly show that the Dab-containing peptides exhibit the most stable α-helical structures, making them strong candidates for biological applications such as antimicrobial or anticancer activities. Orn-containing peptides lack stable secondary structure in water, suggesting they may require membrane-mimicking environments to adopt a functional conformation.

Dap-containing peptides have intermediate properties, potentially limiting their effectiveness compared to Dab-containing analogs. Aurein 1.2 remains a benchmark peptide due to its strong helical structure and stability.

## Figures and Tables

**Table 1:** Structure and analytical data for newly synthesized compounds.

Code	Structure	Molecular Formula	MM Exact	[M+H] ^+^ Found	RT (min)	M.P (°C)	α ^20^_546_ (°) *
Aurein 1.2	Gly^1^-Leu^2^-Phe^3^-Asp^4^-Ile^5^-Ile^6^-Lys^7^-Lys^8^-Ile^9^-Ala^10^-Glu^11^-Ser^12^-Phe^13^-NH_2_	C_71_H_114_N_16_O_18_	1479.76	1480.10	2.013	242.0	−28
EH [Orn]^7^	Gly-Leu-Phe-Asp-Ile-Ile-Orn^7^-Lys-Ile-Ala-Glu-Ser-Phe-NH_2_	C_70_H_112_N_16_O_18_	1465.73	1466.47	7.09	233.4	−10
EH [Orn]^8^	Gly-Leu-Phe-Asp-Ile-Ile-Lys-Orn^8^-Ile-Ala-Glu-Ser-Phe-NH_2_	C_70_H_112_N_16_O_18_	1465.73	1466.84	8.45	234.1	−8
EH [Orn]^7,8^	Gly-Leu-Phe-Asp-Ile-Ile-Orn^7^-Orn^8^-Ile-Ala-Glu-Ser-Phe-NH_2_	C_69_H_110_N_16_O_18_	1451.71	1452.86	7.21	212.3	−16
EH [Dab]^7^	Gly-Leu-Phe-Asp-Ile-Ile-Dab^7^-Lys-Ile-Ala-Glu-Ser-Phe-NH_2_	C_69_H_110_N_16_O_18_	1451.70	1452.86	7.07	238.5	24
EH [Dab]^8^	Gly-Leu-Phe-Asp-Ile-Ile-Lys-Dab^8^-Ile-Ala-Glu-Ser-Phe-NH_2_	C_69_H_110_N_16_O_18_	1451.70	1452.86	7.22	250.8	−50
EH [Dab]^7,8^	Gly-Leu-Phe-Asp-Ile-Ile-Dab^7^-Dab^8^-Ile-Ala-Glu-Ser-Phe-NH_2_	C_67_H_106_N_16_O_18_	1423.65	1424.83	7.20	243.9	14
EH [Dap]^7,8^	Gly-Leu-Phe-Asp-Ile-Ile-Dap^7^-Dap^8^-Ile-Ala-Glu-Ser-Phe-NH_2_	C_65_H_102_N_16_O_18_	1394.71	1395.76	7.86	240.0	−26

* Methanol (c = 1).

**Table 2:** The calculated energies of the aurein 1.2 and its analogs and their RMSD from the aurein 1.2 structure.

Code	Calculated Energy of the Generated Structure, kJ/mol	RMSD, Å
Aurein 1.2	293.48	-
EH [Orn]^7^	38.25	0.152
EH [Orn]^8^	311.40	0.268
EH [Orn]^7,8^	294.03	0.381
EH [Dab]^7^	249.32	0.371
EH [Dab]^8^	120.72	0.586
EH [Dab]^7,8^	276.15	0.528
EH [Dap]^7,8^	466.28	0.365

**Table 3:** Cytotoxicity of peptides in BALB 3T3 cells, values of CC_50_ and Photo Irritation Factor.

Compounds	Mean CC_50_ ± SD (µM)		PIF *
	−Irr	+Irr **	
Aurein 1.2	87.59 ± 2.45	87.22 ± 1.88	1
EH [Orn]^7^	133.65 ± 7.4	102.50 ± 7.40	1.30
EH [Orn]^8^	75.62 ± 3.33	59.45 ± 5.21	1.27
EH [Orn]^7,8^	157.25 ± 6.38	101.25 ± 3.70	1.55
EH [Dab]^7^	158.29 ± 4.31	113.30 ± 5.57	1.40
EH [Dab]^8^	92.08 ± 1.97	81.38 ± 2.34	1.13
EH [Dab]^7,8^	201.81 ± 10.11	181.11 ± 1.81	1.11
EH [Dap]^7,8^	360.00 ± 2.52	357.46 ± 3.03	1.01
Chlorpromazine ***	12.37 ± 0.93	2.04 ± 0.07	6.06

* PIF—Photo Irritation Factor: PIF < 2 = not phototoxic. 2 < PIF < 5 = possible phototoxicity. PIF > 5 phototoxic. ** Irr—Irradiation. *** Chlorpromazine (positive control).

**Table 4:** Average IC_50_ values and selectivity index (SI).

Compounds	Mean IC_50_ ± SD (µM)			SI *	
	MCF-12F	MCF-7	MDA-MB-231	MCF-7	MDA-MB-231
Aurein 1.2	71.37 ± 2.88	68.87 ± 2.12	70.93 ± 2.06	1.04	1.01
EH [Orn]^7^	100.70 ± 5.25	80.51 ± 1.55	134.56 ± 3.94	1.25	0.75
EH [Orn]^8^	44.33 ± 0.52	44.38 ± 1.08	44.85 ± 0.71	1.00	0.99
EH [Orn]^7,8^	112.12 ± 7.14	88.93 ± 3.14	122.70 ± 6.47	1.26	0.91
EH [Dab]^7^	92.07 ± 2.12	74.39 ± 3.29	124.91 ± 4.97	1.24	0.74
EH [Dab]^8^	68.31 ± 3.51	67.21 ± 2.57	79.74 ± 1.78	1.02	0.86
EH [Dab]^7,8^	167.93 ± 2.54	92.23 ± 1.80	163.82 ± 2.51	1.82	1.03
EH [Dap]^7,8^	307.96 ± 21.71	288.65 ± 19.83	372.21 ± 3.17	1.07	0.83
Doxorubicin ***	0.601 ± 0.059	0.68 ± 0.031	2.528 ± 0.11	0.88	0.237

* Selectivity index (SI), SI = IC_50_ (normal cells)/IC_50_ (tumor cells). *** Doxorubicin (positive control).

**Table 5:** MIC and MBC values of Aurein 1.2 analogs against *B. subtilis* NBIMCC 3562 and *E. coli* NBIMCC K12 407.

Peptides	*Bacillus subtilis* NBIMCC 3562		*Escherichia coli* NBIMCC K12 407	
	MIC, [µg/mL]	MBC, [µg/mL]	MIC, [µg/mL]	MBC, [µg/mL]
Aurein 1.2	160	NI	40	NI
EH [Orn]^7^	80	320	80	NI
EH [Orn]^8^	40	NI	40	NI
EH [Orn]^7,8^	80	320	80	NI
EH [Dab]^7^	80	320	80	NI
EH [Dab]^8^	80	320	80	NI
EH [Dab]^7,8^	160	NI	160	NI
EH [Dap]^7,8^	320	NI	NI	NI

NI—no inhibition monitored.

## Data Availability

The data presented in this study are available from the authors.
